# MicroRNA-155 at the crossroads of inflammation and vascular dysfunction in essential hypertension

**DOI:** 10.1007/s11033-026-12039-8

**Published:** 2026-05-30

**Authors:** Norizam Salamt, Nur Aishah Che Roos, Nik Noorul Shakira Mohamed Shakrin, Azizah Ugusman

**Affiliations:** 1 Pharmacology Unit, Faculty of Medicine and Defence Health, National Defence University of Malaysia, Kuala Lumpur, Malaysia; 2 Medical Microbiology and Immunology Unit, Faculty of Medicine and Defence Health, National Defence University of Malaysia, Kuala Lumpur, Malaysia; 3 Tropicalisation Cluster, Research Centre for Defence and Security Science and Technology, Sultan Ibrahim Centre of Excellence for Research and Innovation (PaKSI), National Defence University of Malaysia, Kuala Lumpur, Malaysia; 4 Department of Physiology, Faculty of Medicine, National University of Malaysia, Kuala Lumpur, Malaysia

**Keywords:** microRNA-155, Hypertension, Inflammation, Endothelial dysfunction

## Abstract

MicroRNA-155 (miR-155) has emerged as a pivotal regulator in the development of hypertension. However, its role in the mechanism underlying hypertension remains incompletely defined, especially in vascular inflammation and dysfunction. This review synthesizes and evaluates the published literature on the role of miR-155 in the regulation of inflammation, endothelial dysfunction (ED), and vascular smooth muscle cells (VSMCs) function in essential hypertension (EH). We evaluated experimental and clinical studies investigating the expression patterns, molecular targets, and pathways involving miR-155 in endothelial cells (EC), VSMCs in hypertensive conditions. MiR-155 is consistently upregulated in the circulating and tissue of hypertensive models and patients. MiR-155 intensifies inflammation by suppressing endothelial nitric oxide synthase (eNOS) production, enhancing nuclear factor kappa-light-chain-enhancer of activated B cells (NF-κB) signaling, and promoting pro-inflammatory cytokine expression. In EC, miR-155 directly targets eNOS, leading to impaired nitric oxide (NO) bioavailability and vasodilation by inhibiting the phosphatidylinositol-3-kinase/serine/threonine kinase (PI3K/Akt) pathway. miR-155 modulates the renin-angiotensin-aldosterone system (RAAS) by directly targeting angiotensin II Type 1 receptor (AT1R) mRNA, reducing its expression and attenuating angiotensin II (Ang II) signaling. In VSMCs, it promotes phenotypic switching and contributes to vascular remodeling through the soluble guanylate cyclase/cyclic guanosine monophosphate (sGC/cGMP) pathway. These coordinated actions foster a pro-inflammatory, vasoconstrictive state that underpins hypertension development. MiR-155 acts as a central molecular link between inflammation and vascular dysfunction in essential hypertension by modulating endothelial function, vascular inflammation, and smooth muscle cell behavior. Its modulation highlights miR-155 as a potential therapeutic target to restore endothelial homeostasis and limit vascular damage in hypertensive patients.

## Introduction

Hypertension is a major risk factor for all cardiovascular morbidity and mortality globally [1]. It is defined by a sustained increase in systolic blood pressure (SBP) (≥ 140 mmHg) and/or diastolic blood pressure (DBP) (≥ 90 mmHg) according to standard guidelines for managing hypertension [2, 3]. The prevalence of hypertension is particularly high in low- and middle-income countries, where an estimated 1.28 billion people are affected, with 46% of them unaware of their condition [4]. Hypertension is categorized into primary and secondary hypertension. Primary hypertension, also known as essential hypertension (EH), accounts for 95% of cases without an identifiable cause [5]. However, several modifiable risk factors can contribute to the increase in blood pressure (BP), including older age, overweight/obesity, sedentary lifestyle, high-salt diet, smoking, and alcohol consumption [1]. Additionally, evidence has shown that genetic factors also play an important role in the development of EH [6]. Whilst techniques such as genome-wide association studies (GWAS) identify genes linked to hypertension, multi-omics profiling (genomics, transcriptomics, and proteomics) provides a more comprehensive understanding of its molecular mechanism.

Within the transcriptomic landscape, microRNAs (miRNAs) have emerged as key post-transcriptional regulators influencing the development and progression of hypertension. Since its discovery in the 1990s, numerous literatures have reported studies on miRNA and hypertension. Because miRNA expression is highly specific to tissues and cell types, they serve as valuable biomarkers and therapeutic targets in various diseases. MiRNAs can regulate up to 60% of coding genes, positioning them as crucial elements for maintaining stability in biological processes [7]. Due to their stability in body fluids and their tissue-specific expression patterns, miRNAs are extensively investigated for disease development and remodeling of the heart [8]. They were reported to be involved in key cardiovascular processes, such as endothelial function, vascular tone, inflammation, and fibrosis, as well as modulating critical pathways such as the renin-angiotensin-aldosterone system (RAAS) and oxidative stress, hence highlighting their potential as therapeutic targets for cardiovascular disorders [9, 10].

Several miRNAs strongly influenced BP regulation and vascular health. They were linked to the pathogenesis of hypertension by modulation of the (1) RAAS pathways (miR-181a, miR-133a), (2) vascular and endothelial functions (miR-143/145, miR-29a-3p/29b-3p, miR-214-3p), (3) oxidative stress and mitochondrial function (miR-21) as well as (4) tubular transport (miR-192-5p, miR-195-5p) [10]. Emerging evidence suggests that miR-155 plays a pivotal role in modulating inflammatory pathways associated with the pathogenesis of hypertension. MiR-155 has been implicated in various inflammatory diseases by activating pro-inflammatory cytokines and immune cell expression [11]. However, its specific contributions to the mechanisms underlying the inflammation and vascular dysfunction in hypertension are poorly defined. For instance, miR-155 may play contradictory roles in endothelial homeostasis, either by promoting endothelial dysfunction (ED) or conferring protection against inflammation [12, 13]. In addition, there is no standardized diagnostic threshold for miR-155, which is critical for distinguishing different hypertensive states [14]. While recent studies have shown that miR-155 holds promise as a diagnostic biomarker for cardiovascular disease and hypertension, its translation into clinical practice remains limited [13, 14]. This knowledge gap hinders the development of miR-155 as a potential diagnostic and prognostic biomarker, as well as a target for therapeutic intervention in hypertension. Elucidating the role of miR-155 in regulating inflammatory pathways, ED, and vascular remodeling is essential for advancing novel therapeutic strategies and improving the management of hypertension and its associated complications.

This review will provide a comprehensive overview of the role of miR-155 in BP regulation, including modulation of the inflammatory pathways and endothelial functions. Additionally, this review will discuss the future directions of this field pertaining to miR-155 and its implications in the clinical setting.

## Methods

A comprehensive literature search was conducted using the following electronic databases: PubMed, Scopus, Web of Science, and Science Direct. Keywords used in the search included “microRNA-155,” “miR-155,” “essential hypertension,” “vascular dysfunction,” “inflammation,” “endothelial dysfunction,” and “vascular smooth muscle cells”. To ensure comprehensive coverage of the topic, both original research articles and review papers were considered. Studies published in English were included, and both animal model studies and human clinical studies were evaluated. Grey literature (e.g., conference proceedings and theses) was excluded. Data extraction included the name of the author and publication year, sample type, targeted molecule, miR-155 regulation, and its findings/clinical correlation are presented in Table 1.

**Table 1:** Data extraction table on miR-155 and hypertension

Author (Year)	Sample type	Targeted molecules	miR-155 regulation	Findings / Clinical Correlation	Ref.
Yan et al. (2025)	Human serum	NA	↑	• MiR-155 positively correlates with SBP, triglycerides, and total cholesterol. • MiR-155 was higher in EH patients who were carriers of the rs767649 TT genotype. • High levels of mir-155 indicate susceptibility to EH.	[12]
Huang et al. (2020)	Human plasma	NA	↑	• MiR-155 positively correlated with SBP and DBP and inflammatory markers (CRP and IL-6). • MiR-155 can distinguish hypertensive patients from the control group.	[13]
Ceolotto 2011	Human PBMC	AT1R	↓	• MiR-155 negatively correlated with AT1R. • AT1R positively correlated with SBP and DBP. • Low miR-155 → ↑AT1R → ↑BP	[14]
Kara 2021	Human plasma	NA	↔	NA	[15]
Huang et al. (2016)	Human Plasma	AT1R	↑	• MiR-155 positively correlated with SBP and DBP. • MiR-155 may be a potential non-invasive biomarker of target organ damage in hypertensive patients.	[16]
DuPont 2016	Rat Mesenteric arteries & human serum	AT1R, LTCC	↓	• MiR-155 negatively regulated by MR. • MR → ↓miR-155 → ↑AT1R & LTCC→ ↑vasoconstriction & ROS	[17]
Sun 2012	HUVEC	eNOS	↑	• MiR-155 negatively correlated with eNOS expression. • ↑miR-155 → ↓eNOS/NO → ↑ED → ↑BP	[18]
Xu 2018	Rat aorta + VSMC	p27	↑	• MiR-155 negatively regulated p27 protein. • ↑miR-155 → ↓p27, ↓α-SMA → ↑VSMC proliferation/remodeling.	[19]
Tong 2023	Rat VSMC	BACH1	↑	• ↑miR-155 → ↓BACH1 → ↓ROS & VSMC migration	[20]

↑increase; ↓decrease; ↔ no change; SBP = systolic blood pressure; DBP = diastolic blood pressure; EH = essential hypertension; CRP = C-reactive protein; IL-6 = interleukin-6; AT1R = angiotensin-I receptor; BP = blood pressure; MR = mineralocorticoid receptor; LTCC = L-type calcium channels; ROS = reactive oxygen species; eNOS = endothelial nitric oxide synthase; NO = nitric oxide; ED = endothelial dysfunction; α-SMA = alpha-smooth muscle actin; BACH1 = BTB and CNC homology 1; VSMC = vascular smooth muscle cells; PBMC = peripheral blood mononuclear cells; HUVEC = human umbilical vein endothelial cells; NA = not applicable

### MicroRNA-155

**Fig. 1:**
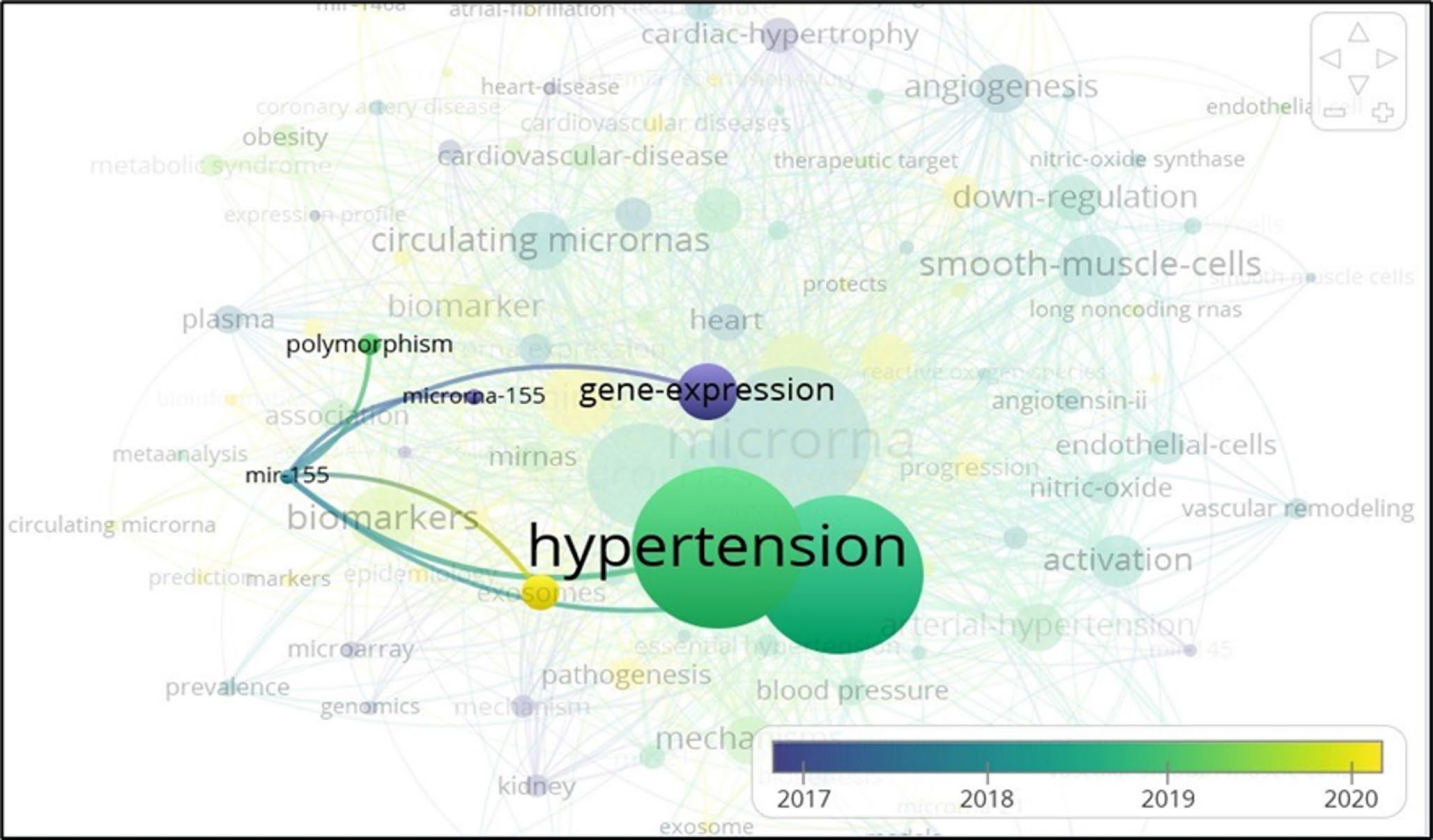
Bibliometric network analysis reveals a strong central cluster linking miR-155 and hypertension

Recently, microRNA-155 (miR-155) has garnered significant attention in the scientific community due to its diverse biological implications [21]. A bibliometric network analysis (Fig. 1) highlights the strong thematic link between miR-155 and hypertension, as well as the recent emergence of “biomarkers” as a key area of study. The size and proximity of these nodes indicate that studies connecting to BP regulation, genetic variation, and circulating diagnostic biomarkers are increasingly prevalent in recent years. Additionally, terms such as “endothelial cells”, “smooth muscle cells”, “angiotensin II”, and “nitric oxide” appear in the surrounding network, suggesting that recent studies on miR-155 are particularly focused on the regulation of vascular inflammation, nitric oxide (NO) signaling, and the renin-angiotensin-aldosterone system (RAAS) pathway in hypertension.

**Fig. 2:**
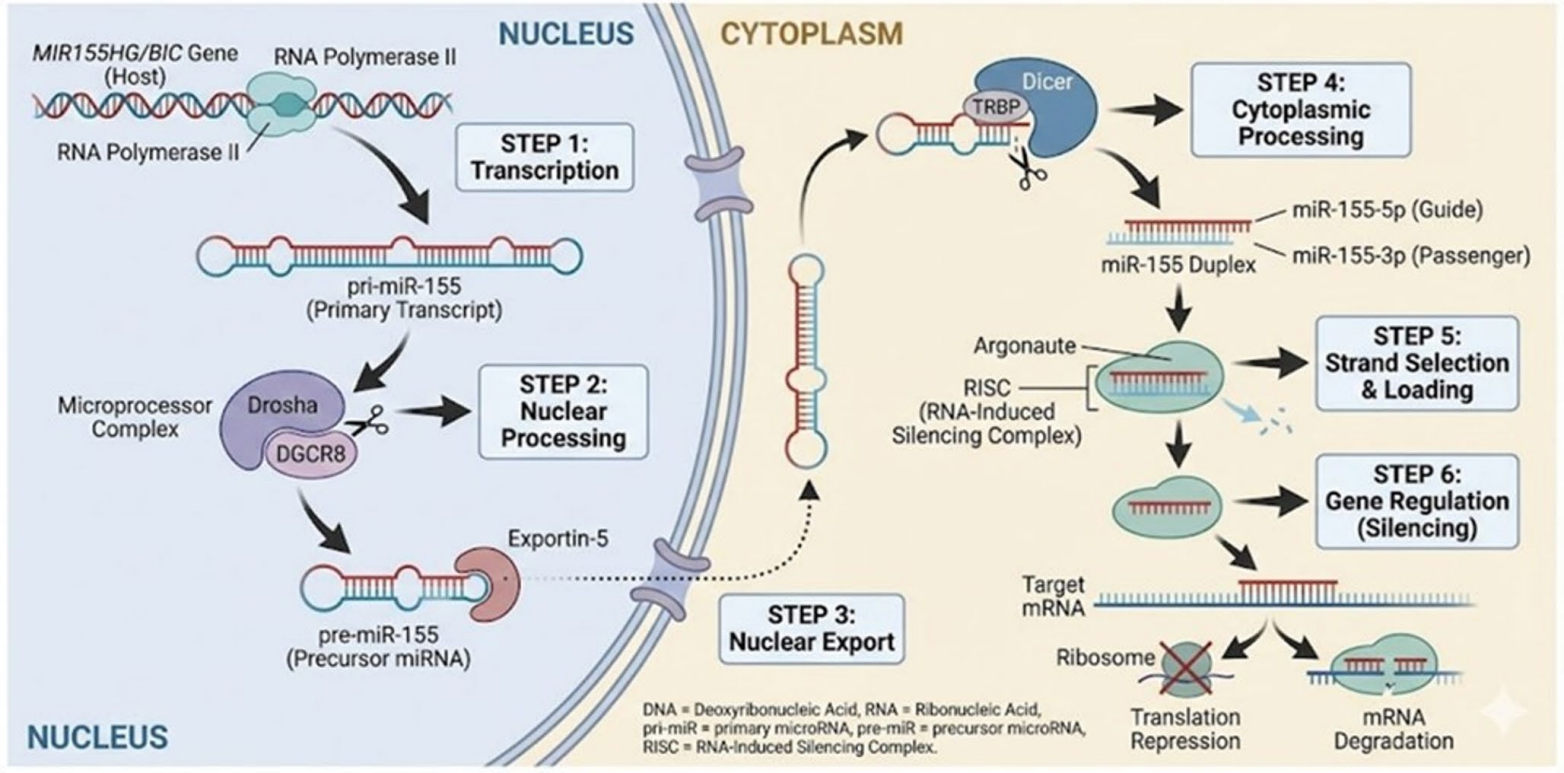
The biogenesis pathway of miR-155. Biologically, miR-155 is transcribed from the B-cell Integration Cluster (BIC), which is located on chromosome 21, and encoded by the *MIR155HG* gene. (Step 1) The process initiates in the nucleus, where RNA Polymerase II transcribes the host gene (*MIR155HG*) into the primary transcript (pri-miR-155). (Step 2) The pri-miR-155 is cleaved by the microprocessor complex (Drosha/DGCR8) into the precursor pre-miR-155, (Step 3) which is exported to the cytoplasm by Exportin-5. (Step 4) In the cytoplasm, the RNase III enzyme Dicer processes the precursor into a duplex. (Step 5) The mature strand is then selected and loaded into the RNA-Induced Silencing Complex (RISC) to guide the repression or degradation of target mRNAs (Step 6)

Biologically, miR-155 is transcribed from the B-cell Integration Cluster (BIC), which is located on chromosome 21, and encoded by the MIR155HG gene. They are mainly expressed in the thymus and spleen [22]. Regarding miRNA-155, it is processed into mature miR-155-5p and miR-155-3p (Fig. 2). Previously, the miR-155-5p form was considered the mature strand (guide strand), as it targets a larger array of transcripts and has higher thermodynamic stability [23]. However, current studies recognize that both miR-155-5p and miR-155-3p are biologically functional miRNAs [24]. Accumulating evidence also indicates that miR-155 exerts regulatory control over immune and inflammatory responses [25].

Recent studies have reported altered circulating and tissue-specific levels of miR-155 in both human hypertensive patients and experimental models, suggesting its potential utility as a biomarker or therapeutic target. Circulating miR-155 is consistently elevated in EH, with significantly higher levels of miR-155 reported in EH compared to white coat hypertension (WCH) patients and the normal BP group [13]. A similar upregulation of miR-155 expression was also observed in hypertensive patients of the Chinese Tibetan population [12]. In contrast, miR-155 expression levels are decreased and negatively correlated with BP in the spontaneous hypertensive rats (SHR) compared to the age-matched Wistar Kyoto (WKY) rats [26]. This difference in the regulation of miRNA expression may be due to the differences in cellular origin and compartment-specific biology. Circulating miR-155 predominantly arises from activated immune cells, particularly monocytes, macrophages, and T-lymphocytes, reflecting systemic inflammation, while aortic miRNA profiles mainly represent endothelial cells (EC) and vascular smooth muscle cells (VSMC), which respond to local mechanical stress [27, 28]. Aortic miRNAs can function as intracellular miRNAs that control local gene expression within aortic tissue and as extracellular miRNAs when released into the extracellular space. On the other hand, circulating miRNAs are extracellular miRNAs which usually contained in particular vesicles such as exosomes. Presence of exosome encapsulating miR-155 protects it from degradation, hence increases miR-155 expression in plasma, and enables circulating miR-155 to act as a long-distance messenger between organs or tissues [29]. The expression pattern of miR-155 in BP dysregulation was linked to its regulatory role in inflammation, endothelial function, and vascular smooth muscle cells (VSMCs) behavior [28].

### MiR-155 and vascular inflammation

Vascular inflammation refers to the inflammatory responses within blood vessels that contribute to the development and progression of high BP [30]. Inflammation can disrupt the normal function of the endothelium, leading to endothelial damage and dysfunction, which eventually worsens blood pressure and increases the risk of cardiovascular disease. This inflammation process is fundamentally driven by the tumor necrosis factor-alpha (TNF-α) signaling cascade that results in increased miR-155. Hypertensive conditions can trigger the release of TNF-α, which then activates the nuclear factor kappa-light-chain-enhancer of activated B cells (NF-kB) signaling pathway and promotes the transcriptional induction of miR-155 [18]. The upregulated miR-155 targets and suppresses endothelial nitric oxide synthase (eNOS) production, reduces NO bioavailability, and leads to vasoconstriction. Simultaneously, miR-155 also enhances the expression of adhesion molecules and pro-inflammatory markers, and eventually causes inflammation [31]. Huang et al. (2020) demonstrated a simultaneous increase in both miR-155 expression and inflammatory markers, such as interleukin-6 (IL-6) and C-reactive protein (CRP), in hypertensive patients [13]. A similar observation was reported in hypertensive rat serum, where inflammatory markers (IL-6 and TNF-α) were elevated, accompanied by concomitant reductions in eNOS and NO [31]. Additionally, overexpression of miR-155 will suppress the inhibitor of NF-κB kinase subunit epsilon (IKBKE), which normally limits NF-kB activity. Its suppression by miR-155 “releases the brake” on NF-kB signaling [32]. This facilitates a positive feedback loop that further stimulates the release of inflammatory markers and triggers chronic inflammation [33]. Inhibition of miR-155 was shown to restore eNOS expression and improve endothelium-dependent vasorelaxation impaired by TNF-α stimulation [18].

Moreover, a hypertensive state may also increase reactive oxygen species (ROS) and trigger the production of miR-155. Once upregulated, miR-155 suppresses the translation of Forkhead box O3 (FOXO3a), a transcription factor that commands the production of antioxidant enzymes like superoxide dismutase 2 (SOD2) and catalase (CAT). The FOXO3a suppression loses the ability of the cell to produce SOD2 and CAT, making the body defenseless against the rising ROS. As miR-155 also represses eNOS, the high oxidative stress “uncouples” any remaining eNOS, causing it to produce more superoxide instead of NO, worsening the oxidative stress [34].

### MiR-155 in endothelial dysfunction

Endothelial dysfunction (ED) is defined as impairment in endothelium-dependent vasodilation, angiogenesis, anti-thrombogenesis, and anti-inflammation [35]. It is the hallmark of hypertension and contributes significantly to the pathogenesis of hypertension [36]. In normal conditions, EC maintain vascular homeostasis by releasing NO, suppressing ROS, and preventing immune adhesion cells [37]. In hypertension, this regulatory balance is disrupted. ED alters the physiological interaction between EC and immune cells, promoting leukocyte adhesion and cytokine infiltration into the vascular wall of EC. This, in turn, impairs endothelial function and further amplifies inflammation, establishing a vicious cycle of ED and vascular inflammation in hypertension [36].

A pivotal mechanism underlying hypertensive ED is the suppression of NO availability. Low levels of NO increase peripheral resistance, causing vasoconstriction and eventually increasing BP levels [38]. Moreover, ROS may also inactivate NO, which will trigger lipid peroxidation and promote oxidative damage to endothelial membranes. Ultimately, it impairs the vascular tone and increases the susceptibility of foam cell formation [39].

MiR-155 has emerged as an important regulator of ED in hypertension. It was found to be highly expressed in hypertensive animal models and hypertensive patients [12, 40]. An increase in miR-155 expression promotes ED by inhibiting the phosphatidylinositol-3-kinase/serine/threonine kinase (PI3K/Akt) signaling pathway, leading to impaired NO synthesis and altered ROS production [35]. Inhibition of PI3K/Akt is found to be associated with reduced eNOS phosphorylation and inhibited NO production [41]. Another study provides corroborative evidence whereby deficiency of miR-155 inhibits PI3K/Akt signaling pathway, hence reduces ROS production while enhances NO generation [35].

NADPH oxidase (NOX), particularly NADPH oxidase 2 (NOX2), is another mechanistic contributor linking miR-155 and ED. NOX2 is shown to regulate the synthesis, stability, and bioavailability of NO and ROS generation [42]. Normally, NOX2 reduces ROS production through its direct ROS scavenging action and NOX inhibition [35]. In hypertension, elevated miR-155 expression increases NOX2 activity and augments ROS generation, further compromising NO bioavailability [43]. Conversely, miR-155 inhibition decreases NOX2 expression and ROS generation, resulting in improved NO synthesis and endothelial homeostasis [44].

ROS can cause damage to cell membranes through lipid peroxidation. Additionally, ROS may disrupt the regulation of vascular tone and increase the likelihood of foam cell formation [39]. Huang et al. (2020) found a significant positive correlation between miR-155 and low-density lipoprotein (LDL) cholesterol in hypertensive patients [13]. Under pathological conditions, elevated levels of oxidized-LDL (ox-LDL) increase oxidative stress, leading to ED, upregulation of several cell surface adhesion molecules, and attracting leukocyte adhesion [45]. Liu et al. (2015) showed that reducing miR-155 levels can decrease ROS production and promote NO generation even when exposed to ox-LDL conditions [35]. In other words, knocking down miR-155 can reduce cell apoptosis, promote cell proliferation, and angiogenesis in both normal and disease states. These effects are believed to be regulated by decreased caspase-3 expression and increased levels of epidermal growth factor receptor (EGFR), extracellular regulated protein kinase (ERK) 1/2, and p38 mitogen-activated protein kinase (MAPK) expression and phosphorylation [35]. ROS production may also cause vasoconstriction in aging rats, where miR-155 was suppressed by high mineralocorticoid receptor (MR) expression. This, in turn, upregulated AT1R and L-type calcium channels (LTCC) expression and enhanced Ang II-induced and LTCC-mediated vasoconstriction and vascular oxidative stress, components of vascular aging [17]. Therefore, the findings suggest that an increased level of miR-155 promotes endothelial injury by impairing NO production, enhances inflammation, and potentially accelerates atherogenesis.

**Fig. 3:**
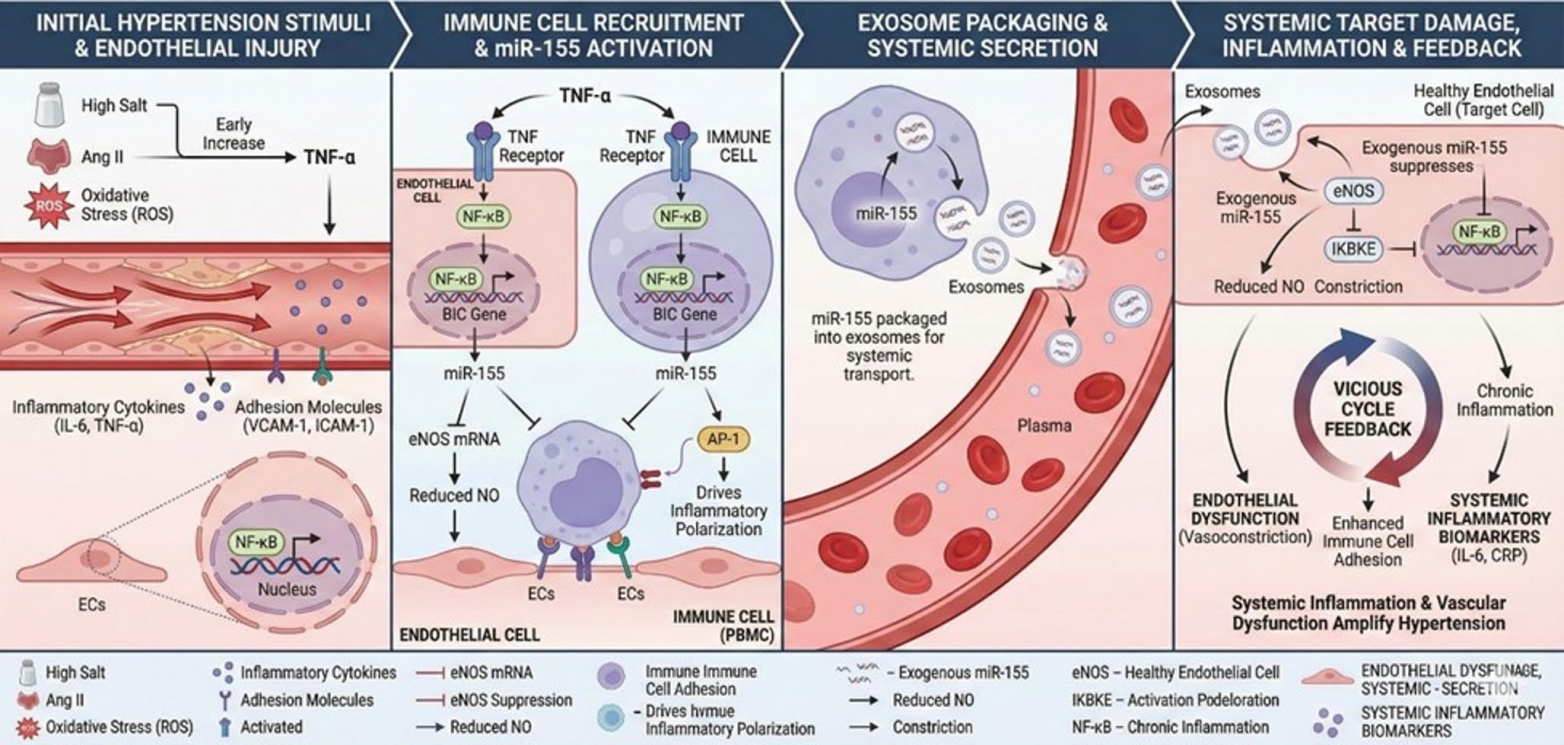
The miR-155 feedback loop in hypertensive-induced inflammation and endothelial dysfunction. (Step 1) Pro-hypertensive stimuli induce the release of TNF-α and adhesion molecules on the endothelium. (Step 2) TNF-α binds to the receptor, activating the NF-kB pathway and driving the robust expression of miR-155. (Step 3) Intracellular miR-155 is packaged into exosomes, which are then secreted into the plasma. (Step 4) These exosomes deliver miR-155 to healthy endothelial cells, where it suppresses eNOS and IKBKE. This dysregulation creates a “vicious cycle” of vasoconstriction and chronic inflammation, further amplifying blood pressure, inflammation, and vascular injury

The miR-155 feedback loop in hypertensive-induced inflammation and endothelial dysfunction are shown in Fig. 3.

### MiR-155 in RAAS regulation

The RAAS is essential in maintaining fluid and electrolyte balance in the regulation of BP [46]. Renin, secreted by granular cells in the juxtaglomerular apparatus of the kidneys, converts angiotensinogen released from the liver to angiotensin I (Ang I) [47]. The angiotensin‑converting enzyme (ACE) then mediates the conversion of Ang I to angiotensin II (Ang II), which subsequently activates the angiotensin receptors [48]. The angiotensin II type 1 receptor (AT1R) mediates the effects implicated in the pathogenesis of hypertension [49]. Stimulation of AT1R by Ang II causes vasoconstriction, sodium and water retention, and activates aldosterone synthesis [49]. Ang II causes vasoconstriction by activating G‑proteins in the vessels, leading to increased peripheral vascular resistance. Ang II also regulates the production of oxidative stress by activating NOX, which impairs NO synthesis and increases BP [50]. In addition, Ang II induces the secretion of aldosterone from the adrenal cortex, resulting in sodium and water retention in the kidneys, and eventually elevates BP [48].

MiR-155 displays functional duality in the regulation of BP, capable of driving both hypertensive injury and compensatory protective mechanisms, by modulating the expression of AT1R, eNOS, and inflammatory cytokine signaling [51]. In a protective mechanism, high expression of miR-155 is negatively associated with AT1R by directly binding to the 3’ UTR of AT1R mRNAs and repressing the AT1R protein in VSMC, thereby decreasing Ang II responsiveness [14]. Eventually, it will reduce major drivers of sustained BP elevation, such as vasoconstriction, vascular hypertrophy, and remodeling [52]. Additionally, miR-155 targets BTB and CNC homology 1 (BACH1) to attenuate oxidative stress and maintain NO bioavailability [20]. Collectively, these vascular effects position miR-155 as a critical physiological brake that attenuates excessive RAAS signaling, limiting hypertensive vascular damage specifically within VSMCs and resistance arteries.

Despite these protective effects, miR-155 also participates in the signaling pathway that can worsen endothelial injury and inflammation in EC. In the presence of hypertensive stimuli, miR-155 is strongly induced by pro-inflammatory factors, such as TNF-α and IL-1β, via activation of NF-kB [53]. This elevated miR-155 inhibits eNOS, reduces NO bioavailability, and thus impairs vasorelaxation. miR-155 further intensifies NF-kB activity by inhibiting IKBKE. Inhibition of IKBKE amplifies inflammatory gene expression, increases adhesion molecule expression, and accelerates leukocyte infiltration into the vascular wall [54]. This inflammatory cascade fosters ED and maintains a vicious cycle where upregulated miR-155 further propagates endothelial inflammation and oxidative stress.

Overall, miR-155 offers anti-remodeling and anti-hypertensive benefits by limiting RAAS signaling and oxidative stress in VSMC. In contrast, sustained overexpression of miR-155 in EC promotes inflammatory signaling, decreases NO production, and worsens ED. Thus, the net impact of miR-155 in hypertension reflects a dynamic equilibrium between its protective inhibition of RAAS signaling and its deleterious promotion of inflammation-driven endothelial injury.

### MiR-155 in vascular smooth muscle cell function

Alterations in VSMCs’ function contribute significantly to the development of hypertension. In hypertension, VSMC often shift from a contractile to a pathological synthetic phenotype, contributing to increased vascular stiffness and peripheral resistance [14]. Experimental and clinical studies have investigated the role of miR-155 in the pathogenesis of hypertension and its relationship with VSMC proliferation [19, 34]. miR-155 was found as a potent regulator of Ang II-induced VSMC proliferation by inhibiting AT1R [55, 57]. Upregulation of miR-155 facilitates phenotypic and functional alterations of VSMC by inhibiting the activity of the soluble guanylyl cyclase A (sGC)/cyclic guanosine monophosphate (cGMP) axis by downregulating soluble guanylate cyclase β1 (sGCβ1) [28]. This is further supported by the downregulation of sGC expression in SHR and Ang II-induced hypertensive mice [56]. In addition, TNF-α and miR-155 mimic treatment also suppressed sGC/cGMP production, which induced VSMC phenotypic switching and attenuated NO-mediated vasodilation [28]. It is shown that miR-155 is a negative regulator of the sGC/cGMP pathway, and thus, disruption of the sGC/cGMP pathway by miR-155 may contribute to the mechanism underlying hypertension [28]. Moreover, a study by Xu et al. (2018) showed that elevated miR-155 increased VSMC proliferation by negatively regulating p27 protein and α-smooth muscle actin (α-SMA), a contractile marker gene for VSMC, in hypertensive rats [19].

### Clinical implications and future directions of miR-155

MiR-155 has emerged as a promising diagnostic and prognostic biomarker for hypertension due to its crucial role in regulating vascular inflammation, ED, and vascular remodeling. Numerous studies demonstrate that miR-155 dysregulation in both hypertensive animal models and patients is associated with ED, endothelial activation, and immune pathway modulation [12, 40]. Elevated miR-155 levels may reflect subclinical vascular pathology prior to the onset of overt symptoms [21], and its dysregulation is linked to a heightened risk of developing hypertension in predisposed individuals [12]. Furthermore, Mehaffey et al. (2017) found a significant correlation between inflammatory markers and hypertension severity [59]. Given that miR-155 expression is influenced by these markers, it could serve as a reliable indicator of an underlying inflammatory state. Notably, circulating miR-155 levels can successfully differentiate hypertensive patients from normotensive controls [13]. Its stability and accessibility in body fluids such as plasma and serum, combined with the availability of standard measurement techniques, position it as an excellent non-invasive tool for early detection and disease monitoring [13, 58].

However, because current evidence is primarily derived from cross-sectional studies, longitudinal clinical trials are required to validate the reliability of miR-155 in predicting the transition from prehypertension to clinical hypertension. Moreover, the viability of miR-155 as a targeted therapeutic intervention warrants extensive investigation to optimize future hypertension management.

## Conclusion

MiR-155 arises as a pivotal regulator in the pathogenesis of EH by modulating endothelial function, vascular inflammation, and VSMC behavior. Through its suppression of eNOS and the sGC/cGMP axis, as well as its regulation of AT1R and inflammatory signaling pathways, miR-155 promotes oxidative stress, ED, and VSMC phenotypic switching. These findings highlight miR-155 as a potential diagnostic or prognostic biomarker for mitigating vascular dysfunction and the progression of hypertension. Genetic variations affecting miR-155 binding sites may influence individual susceptibility to hypertension. Future research should focus on elucidating the precise mechanisms of miR-155 in BP regulation and exploring its potential as a diagnostic or prognostic biomarker.

## Data Availability

No datasets were generated or analysed during the current study.

## Abbreviations

miR-155: microRNA-155
EH: Essential hypertension
ED: Endothelial dysfunction
VSMCs: Vascular smooth muscle cells
ECs: Endothelial cells
eNOS: Endothelial nitric oxide synthase
NO: Nitric oxide
RAAS: Renin–angiotensin–aldosterone system
PI3K/AKT: Phosphoinositide 3-kinase/protein kinase B
AT1R: Angiotensin II type 1 receptor
Ang II: Angiotensin II
NF-κB: Nuclear factor kappa B
